# 5-Aza-2′-Deoxycytidine Alters the Methylation Profile of Bortezomib-Resistant U266 Multiple Myeloma Cells and Affects Their Proliferative Potential

**DOI:** 10.3390/ijms242316780

**Published:** 2023-11-26

**Authors:** Karolina Łuczkowska, Piotr Kulig, Klaudia Rusińska, Bartłomiej Baumert, Bogusław Machaliński

**Affiliations:** 1 Department of General Pathology, Pomeranian Medical University, 70-111 Szczecin, Poland; karolina.luczkowska@pum.edu.pl (K.Ł.); piotrkulig@interia.eu (P.K.); rusinskaklaudia@gmail.com (K.R.); 2 Department of Hematology and Transplantology, Pomeranian Medical University, 71-252 Szczecin, Poland

**Keywords:** multiple myeloma, bortezomib, resistance, methylation inhibitor, 5-Aza-2′-deoxycytidine

## Abstract

Multiple myeloma (MM) is a plasma cell malignancy that accounts for 1% of all cancers and is the second-most-common hematological neoplasm. Bortezomib (BTZ) is a proteasome inhibitor widely implemented in the treatment of MM alone or in combination with other agents. The development of resistance to chemotherapy is one of the greatest challenges of modern oncology. Therefore, it is crucial to discover and implement new adjuvant therapies that can bypass therapeutic resistance. In this paper, we investigated the in vitro effect of methylation inhibitor 5-Aza-2′-deoxycytidine on the proliferative potential of MM cells and the development of resistance to BTZ. We demonstrate that alterations in the DNA methylation profile are associated with BTZ resistance. Moreover, the addition of methylation inhibitor 5-Aza-2′-deoxycytidine to BTZ-resistant MM cells led to a reduction in the proliferation of the BTZ-resistant phenotype, resulting in the restoration of sensitivity to BTZ. However, further in vitro and ex vivo studies are required before adjuvant therapy can be incorporated into existing treatment regimens.

## 1. Introduction

Multiple myeloma (MM) is the second-most-common hematological malignancy, with a complex and multifaceted pathogenesis. MM affects a significant number of people worldwide, with an age-standardized rate of 1.78 per 100,000 individuals in 2020 [1]. It particularly affects patients over 65 years of age but may also occur in younger individuals [2]. The severity of this medical condition is significant, but progress in pharmacotherapy and a profound comprehension of molecular mechanisms have improved the overall prognosis and survival. B lymphocytes transformed into antibody-producing plasma cells are involved in the pathogenesis of MM. Pathologically altered plasma cells have a proclivity for monoclonal, irreversible, and uncontrolled proliferation. This leads to the destruction and impaired functioning of bone marrow and even end-stage organ damage [3]. A complex array of genetic and epigenetic changes, chromosomal aberrations, and angiogenesis disorders plays a crucial role in the pathogenesis of MM. Cytogenetic abnormalities such as del(17p), t(4;14), t(14;16), t(14;20), 1q gain, and p53 are associated with poor prognosis and high-risk MM [4]. Radiation and exposure to certain chemical substances, i.e., asbestos or benzene, are considered risk factors for MM [5]. MM develops from a premalignant condition known as monoclonal gammopathy of undetermined significance (MGUS) or smoldering myeloma (SMM). MGUS is mostly asymptomatic, discovered accidentally, and manifests itself especially in laboratory tests. MGUS progresses to MM with a risk of 1% per year. SMM is a transitional stage between MGUS and MM, with a 10% risk of progression per year [6,7,8,9]. MM patients mainly present with fatigue and bone pain. About 75% of patients suffer from anemia, which contributes to fatigue, and about 80% exhibit osteolytic changes in the skeleton. Although pathognomonic, hypercalcemia (15%) and renal failure (20%) are slightly less common [10]. The treatment of MM continues to be a challenge for hematologists. Despite significant progress in the pharmacotherapy of MM, which has resulted in prolonged survival, the vast majority of patients experience a relapse of the disease and cannot be cured. The most acclaimed drugs used in the treatment of MM patients are alkylating agents (melphalan and cyclophosphamide), corticosteroids, immunomodulatory drugs (IMiDs (thalidomide, lenalidomide, pomalidomide)), and proteasome inhibitors (bortezomib, carfilzomib, and ixazomib) [11]. Bortezomib (BTZ) is a first-generation proteasome inhibitor and a highly effective agent against MM [12]. BTZ inhibits the ubiquitin–proteasome catalytic pathway in cells by directly binding to the 20S proteasome complex [13]. Such interference leads to the accumulation of misfolded and otherwise defective proteins and induces MM cell death [14,15]. Despite the satisfactory effects of BTZ therapy, many patients acquire resistance. The development of resistance is often associated with disease relapse. Several mechanisms contribute to therapy failure, such as abnormal drug transport, activation of detoxification systems, changes in drug targets, cell cycle or apoptosis dominance, and the distortion of signaling pathways [16]. The abovementioned factors lead to increased BTZ excretion, interfere with the formation of the BTZ–proteasome complex and impair the therapeutic response. The development of resistance to BTZ is the basis for the search for new therapeutic targets or mechanisms to suppress resistance.

Epigenetics deals with mechanisms regulating gene expression that do not result from changes in the DNA sequence. It includes DNA methylation, histone modifications, chromatin-regulating proteins in cells, and non-coding RNA [16,17,18]. There are several studies reporting methylation changes associated with chemotherapy resistance in solid tumors. For example, the hypermethylated TGBI promoter in breast cancer is associated with trastuzumab resistance in HER2+ patients [19]. Alterations in methylome were shown to mediate resistance to IMiDs in MM. Cereblon (CRBN) enhancer methylation inhibits CRBN expression, which confers resistance to IMiDs. Moreover, in in vitro studies investigating DNA methyltransferase inhibitors (DNTMis), sensitization to lenalidomide treatment was demonstrated in two MM cell lines [20]. DNMT inhibitors are becoming increasingly attractive therapeutic agents. Azacytidine (AZA) and decitabine are the most successful epigenetic drugs used in in vitro studies of MM therapy [21].

We also investigated the role of epigenetic alterations in the development of BTZ resistance. In one of our previous studies, we established a BTZ-resistant neuroblastoma cell line. Subsequently, we analyzed the methylome of both resistant and sensitive cells and demonstrated that changes in the methylation profile contribute to the development of resistance to this compound [22]. Similarly, in another study, we focused on MM cell lines. First, we established the BTZ-resistant U266 MM cell line (cells were cocultured with BTZ; three repeated treatments were required to obtain the BTZ-resistant phenotype); then, implementing next-generation sequencing, we investigated the mechanisms underlying the development of BTZ resistance. In parallel, we demonstrated the contribution of oxidative phosphorylation and the role of SNORD-family genes that mediate epigenetic changes. Moreover, we showed that vitamins D and K act synergically with BTZ [23]. Therefore, we hypothesized that, first, changes in methylome mediate resistance to BTZ and, second, epigenetic agents such as AZA may act synergistically with BTZ and even restore sensitivity to this compound.

The aim of this study was to investigate alterations in methylome affecting the proliferative potential of MM cells associated with the development of resistance to BTZ. In our research, we focused on methylation, which is crucial for the gene regulation process, including both activation and suppression [24]. It is worth emphasizing that methylation is a reversible process and serves as a starting point in therapy. The hypermethylation of gene promoters may result in the suppression of individual genes, potentially causing the development of multidrug resistance (MDR) [25].

It has been shown several times that resistance to BTZ results from epigenetic alterations, among other causes [23,26]. Therefore, we investigated the impact of changes in the DNA methylation profile on the development of this phenotype. Furthermore, we examined the effect of a methylation inhibitor on the proliferative potential of BTZ-resistant MM cells.

## 2. Results

### 2.1. Proliferation Assay

**Figure 1:**
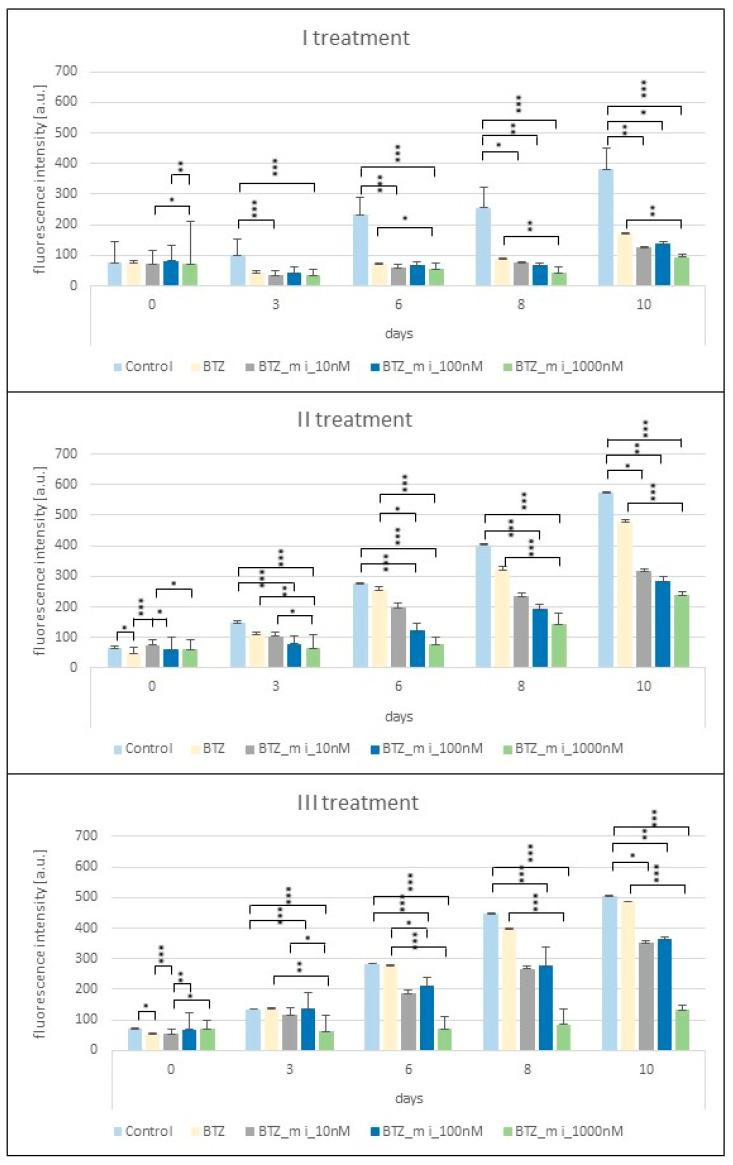
Graphs showing the level of proliferation of U266 cells after each treatment with BTZ and/or a methylation inhibitor (BTZ_m i). Data were not normally distributed; therefore, differences between the groups were analyzed with a Kruskal–Wallis test followed by a post-hoc Dunn test with Bonferroni correction for multiple testing. *p* < 0.05 was considered statistically significant; * *p* < 0.05, ** *p* < 0.01, *** *p* < 0.001.

Analysis of the proliferation results showed a fully BTZ-resistant phenotype after the third treatment of U266 cells. BTZ-resistant cells showed a similar proliferation rate to that of control cells; therefore, we can consider them resistant to BTZ (control = 506.3, SD ± 28.4; BTZ = 486.03, SD ± 52.8) (Figure 1). U266 cells were treated simultaneously with BTZ and the methylation inhibitor to examine whether its use would inhibit the development of a resistant phenotype. The obtained results clearly show a decrease in proliferation with increasing dose of the methylation inhibitor. After the first treatment, when the phenotype of U266 cells was not yet resistant to BTZ, the differences in the degree of proliferation reached several percent when comparing different doses of 5-Aza-2′-deoxycytidine to each other. However, the lowest level of proliferation was already observed at the highest dose of 5-Aza-2′-deoxycytidine. After the second treatment, a decreasing level of proliferation was clearly observed after the use of 5-Aza-2′-deoxycytidine in relation to both control cells and cells treated with BTZ alone. Additionally, a dose of 5-Aza-2′-deoxycytidine 1000 nM most effectively slowed down cell proliferation. Proliferation on day 10 was 62.2% lower compared to proliferation at the remaining doses.

Three treatments of myeloma cells with 5-Aza-2′-deoxycytidine at a dose of 1000 nM reduced the proliferation of BTZ-resistant cells by approximately 72% (*p* < 0.0004) (Figure 1). Based on the obtained results, a dose of 1000 nM 5-Aza-2′-deoxycytidine was selected for further procedures.

### 2.2. Effect of a Methylation Inhibitor on the Development of BTZ Resistance in U266 Myeloma Cells

Methylation analysis provided data immediately after the second (BTZ_m_i_2 and BTZ_2) and third (BTZ_m_i_3 and BTZ_3) treatments of U266 cells. In addition, we performed an analysis 10 days after the third treatment (BTZ_m_i_10d_3) (nothing was added to the medium at that time) to demonstrate whether the resulting changes in methylation levels were permanent and transmitted to daughter cells despite treatment discontinuation. No significant changes in the methylation profile were observed after the first treatment. A detailed analysis of the methylation profile is presented below.

### 2.3. Results of DNA Methylation Profile Analysis Obtained after Two Treatments

**Figure 2:**
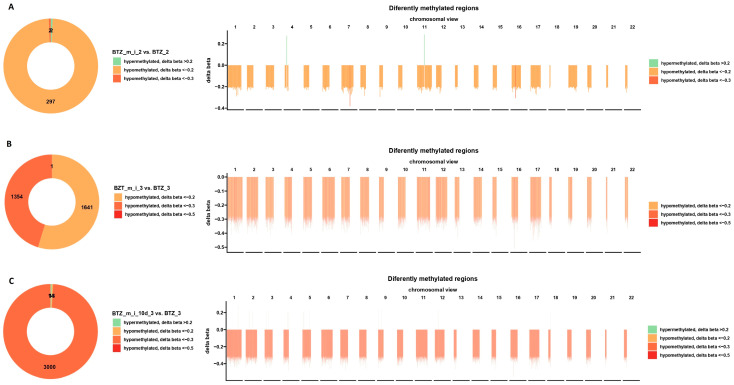
Graphs showing the DNA methylation profile of U266 myeloma cells after two (**A**) and three (**B**) simultaneous treatments with BTZ and a methylation inhibitor (BTZ_m_i) compared to cells treated with BTZ alone (BTZ). In addition, (**C**) shows the DNA methylation profile 10 days after the last treatment. Circular charts show differences in methylation levels in BTZ- and methylation-inhibitor-treated U266 cells relative to cells treated with BTZ (*p* < 0.05). In each section, the distribution of DNA methylation changes on individual chromosomes is presented (orange indicates hypomethylation, and green hypermethylation; *p* < 0.05). BTZ—bortezomib; BTZ_2—bortezomib, second treatment; BTZ_m_i—bortezomib and methylation inhibitor; BTZ_m_i_2—bortezomib and methylation inhibitor, second treatment; BTZ_3—bortezomib, third treatment; BTZ_m_i_3—bortezomib and methylation inhibitor, third treatment; BTZ_m_i_10d_3—bortezomib and methylation inhibitor 10 days after third treatment.

Bioinformatics analysis showed 301 sites (299 hypomethylated and 2 hypermethylated) with altered methylation in myeloma cells treated twice with BTZ and a methylation inhibitor compared to cells treated twice with BTZ alone (Figure 2A). The beta delta values shown in the graphs were obtained by calculating the ratio of the normalized fluorescence intensity values of the probe between the methylated and unmethylated signals (0 = fully unmethylated; 1 = fully methylated). The distribution of methylation changes is shown on each chromosome separately in Figure 2A. Hypermethylation was only observed on chromosomes 4 and 11.

Moreover, bioinformatics analysis showed 2996 sites with altered methylation (only hypomethylated) in myeloma cells treated three times with BTZ and a methylation inhibitor compared to cells treated three times with BTZ alone (Figure 2B). The number of changes in the methylation level increased by almost 10-fold after the third treatment compared to the results obtained after the second treatment (second treatment: 301 changed sites vs. third treatment: 2996 changed sites).

**Figure 3:**
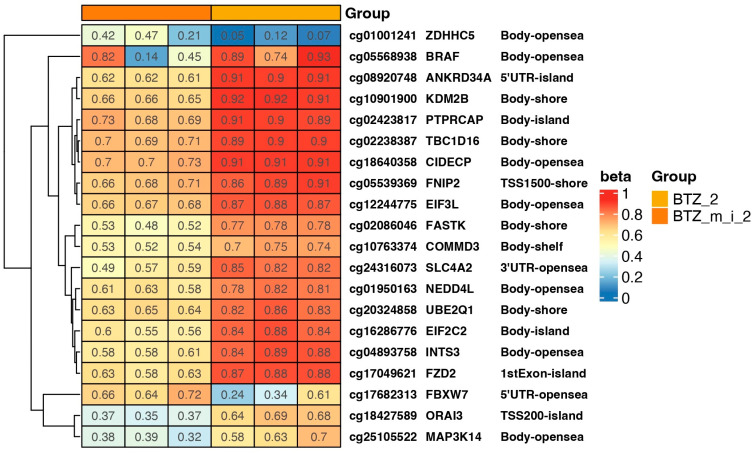
Heat map showing beta values representing the methylation level of selected genes in cells treated twice with BTZ (BTZ_2) and with BTZ and a methylation inhibitor (BTZ_m_i_2). Beta values of “1” indicate full methylation (red), and “0” indicates no methylation (blue) (*p* < 0.05). Gene symbols and methylation sites are marked on the heat map. The heatmap results were visualized using R library ComplexHeatmap. Island—CpG island; 5′UTR—5′ untranslated region; shore—the 2 kb sequences directly up- and downstream of CpG islands; shelf—the 2 kb sequences directly adjacent to the shore; opensea—outside of the shelf region.

Changes in methylation after double treatment of U266 cells with BTZ (BTZ_2) or BTZ and a methylation inhibitor (BTZ_m_i_2) in selected genes are shown in the heat map (Figure 3). The most interesting changes in methylation from the point of view of resistance development were observed in the following genes: FBXW7, ORAI3, and TBC1D16. It is also worth mentioning genes that regulate proliferation processes (ZDHHC5) and epigenetic modifications (KDM2B), as well as those that act as a transcription factor (COMMD3). The observed changes in these genes may significantly influence the development of resistance to BTZ.

**Figure 4:**
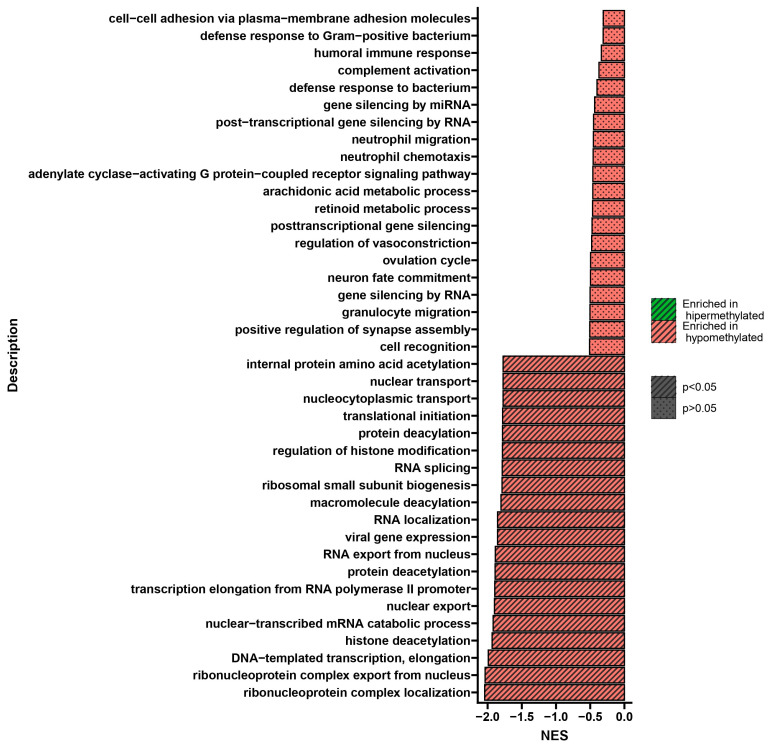
GSEA shows NES (normalized enrichment score) values indicating changes in DNA methylation in genes involved in the regulation of the listed biological processes in U266 cells treated twice with BTZ and a methylation inhibitor compared to cells treated twice with BTZ alone. Red indicates hypomethylation, and green indicates hypermethylation.

Gene set enrichment analysis (GSEA) performed after double treatment of U266 cells with BTZ and a methylation inhibitor compared to cells treated twice with BTZ alone showed process changes correlated only with hypomethylation (Figure 4). GSEA allowed for the isolation of 20 statistically significant processes whose genes showed reduced methylation levels in cells treated with BTZ and a methylation inhibitor compared to cells treated with BTZ alone. The most interesting, statistically significant processes (*p* < 0.05) seem to be RNA splicing and epigenetic alterations, such as histone deacetylation, protein deacetylation, regulation of histone modification, and histone deacetylation, which are important in considering the impact of epigenetic modifications on the development of BTZ resistance.

### 2.4. Results of DNA Methylation Profile Analysis Obtained after Three Treatments

Bioinformatics analysis showed 2996 sites with altered methylation (only hypomethylated) in myeloma cells treated three times with BTZ and a methylation inhibitor compared to cells treated three times with BTZ alone (Figure 2B). The number of changes in the methylation level increased by almost 10-fold after the third treatment compared to the results obtained after the second treatment (second treatment: 301 changed sites vs. third treatment: 2996 changed sites).

**Figure 5:**
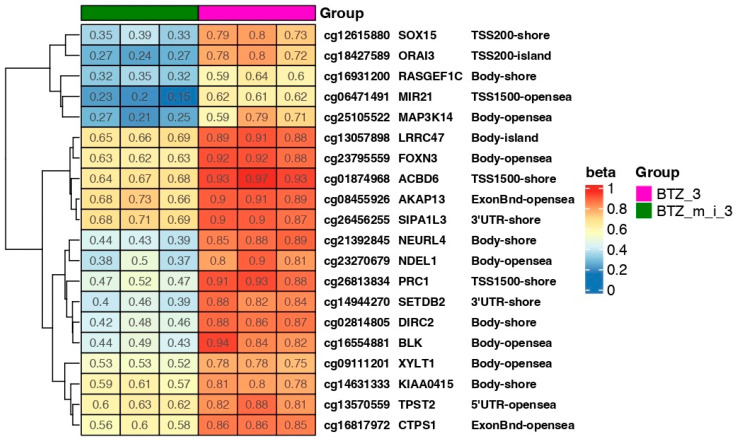
Heat map showing beta values representing the methylation levels of selected genes in cells treated three times with BTZ (BTZ_3) or BTZ and a methylation inhibitor (BTZ_m_i_3). Beta values of “1” indicate full methylation (red), and “0” indicates no methylation (blue) (*p* < 0.05). Gene symbols and methylation sites are marked on the heat map. The heatmap results were visualized using R library ComplexHeatmap). Island—CpG island; 5′UTR—5′ untranslated region; shore—the 2 kb sequences directly up- and downstream of CpG islands; shelf—the 2 kb sequences directly adjacent to the shore; opensea—outside of the shelf region.

Analysis of changes in methylation levels in individual genes after three treatments with BTZ and a methylation inhibitor relative to cells treated with BTZ alone showed hypomethylation of the following genes: MIR21, PRC1, AKAP13, and ORAI3 (Figure 5), which are directly related to the development of drug resistance.

**Figure 6:**
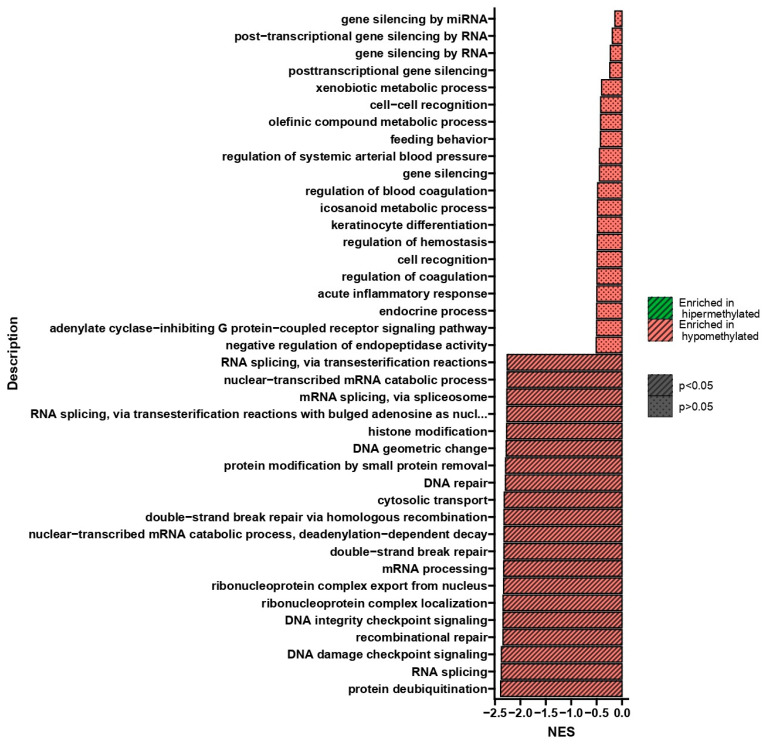
GSEA shows NES values indicating changes in DNA methylation in genes involved in the regulation of the listed biological processes in U266 cells treated three times with BTZ and a methylation inhibitor compared to cells treated three times with BTZ alone. Red indicates hypomethylation, and green indicates hypermethylation.

GSEA performed after the third treatment of U266 cells with BTZ and a methylation inhibitor compared to cells treated three times with BTZ alone showed process changes correlated only with hypomethylation (*p* < 0.05) (Figure 6). The process of RNA splicing plays a very important role in the development of drug resistance. The formation of abnormal splice variants or splicing machinery disorders may cause the development of drug resistance and promote the development of cancer [27]. This process deepens with subsequent treatments (second-treatment NES = −1.78 *p* = 3.4 × 10^−15^; third-treatment NES = −2.37; *p* = 8.2 × 10^−23^) (Figure 4 and Figure 6). Other processes that drew our attention during the GSEA analysis concern DNA damage and repair processes. These processes participate in the development of resistance through the DNA modifications, which allow cancer cells to survive in environments with high levels of genotoxic stress provided by the therapy [28,29]. These biological processes were also identified 10 days after the last treatment, confirming both the importance of these changes in the process of BTZ resistance development and their persistence and transmission to daughter cells.

### 2.5. Results of DNA Methylation Profile Analysis Obtained 10 Days after the Third Treatment

**Figure 7:**
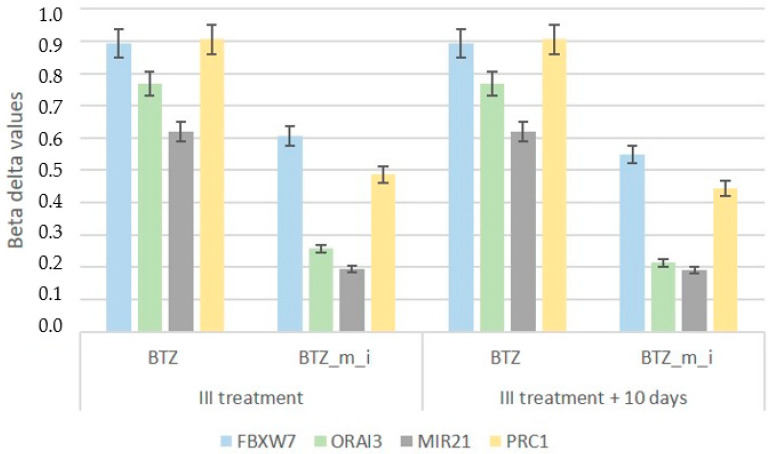
Beta delta values (mean *n* = 3; *p* < 0.05 for all results) for selected genes relevant to the development of BTZ resistance measured immediately and 10 days after the third treatment. BTZ—bortezomib; BTZ_m_i—bortezomib combined with a methylation inhibitor.

**Figure 8:**
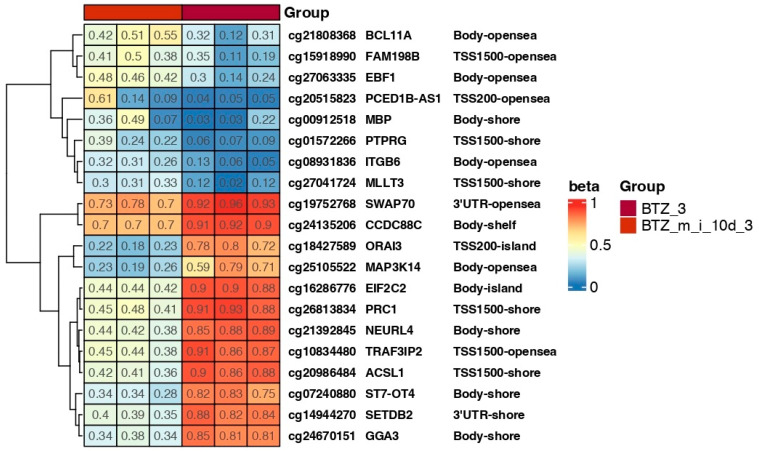
Heat map showing beta values representing the methylation level of selected genes in cells treated three times with BTZ (BTZ_3) or BTZ and a methylation inhibitor 10 days after the third treatment (BTZ_m_i_3). Beta values of “1” indicate full methylation (red), and “0” indicates no methylation (blue) (*p* < 0.05). Gene symbols and methylation sites are marked on the heat map. The heatmap results were visualized using R library ComplexHeatmap. Island—CpG island; 5′UTR—5′ untranslated region; shore—the 2 kb sequences directly up- and downstream of CpG islands; shelf—the 2 kb sequences directly adjacent to the shore; opensea—outside of the shelf region.

**Figure 9:**
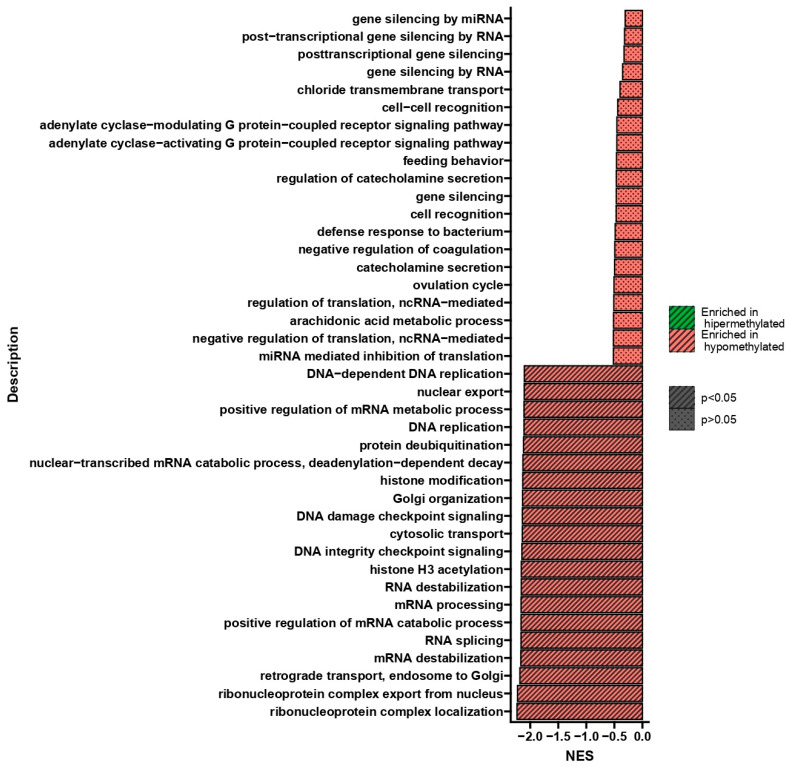
GSEA shows NES values indicating changes in DNA methylation in genes involved in the regulation of the listed biological processes in U266 cells 10 days after the third treatment of cells with both BTZ and a methylation inhibitor compared to cells treated three times with BTZ alone. Red indicates hypomethylation, and green indicates hypermethylation.

Three 24 h treatments of cells with BTZ and a methylation inhibitor induced permanent changes in a large number of genes, especially in the case of genes responsible for the development of resistance, such as FBXW7, ORAI3, MIR21, and PRC1 (Figure 7 and Figure 8). Analysis of the data presented in Figure 7, which shows the beta values of the selected genes immediately after the third treatment and 10 days later, showed comparable levels of methylation at both time points. In addition, a bioinformatics analysis was performed comparing the results of the DNA methylation level immediately after three treatments (BTZ_m_i) vs. 10 days later (BTZ_m_i_10days). The analysis did not show significant changes in DNA methylation. This confirms that the induced changes in DNA methylation are permanent and transmitted to daughter cells despite the withdrawal of factors. There was no enrichment in methylation according to GSEA analysis (Figure 9). 

Bioinformatics analysis conducted 10 days after the third treatment of cells with BTZ and a methylation inhibitor compared to cells treated with BTZ alone revealed 3023 (3009 hypomethylated and 14 hypermethylated) altered methylation sites (Figure 2C). These results are slightly different from those obtained immediately after the third treatment. Areas of hypermethylation were observed on chromosomes 1–5, 9, 12, and 18 (Figure 2C).

## 3. Discussion

Although MM remains an incurable disease, the clinical outcomes of MM patients have improved significantly over time with the development and implementation of various chemotherapy regimens. BTZ is a proteasome inhibitor that is an essential component of various anti-MM treatment regimens. Initially, it was successfully applied in a monotherapy [29,30]. Currently, BTZ is administered in combination with other molecules, e.g., monoclonal antibodies [31,32] or immunomodulatory drugs such as thalidomide [33], lenalidomide [34], and pomalidomide [35]. Moreover, there are treatment regimens consisting of more than three drugs, and often, BTZ is one of the basic ingredients, as in the D-VTd protocol (daratumumab, bortezomib, thalidomide, and dexamethasone) [36]. As mentioned above, BTZ is widely used to treat MM. It can be hypothesized that almost every MM patient will be exposed to BTZ during treatment. Exposure to BTZ exerts environmental pressure on malignant plasma cells, which, in turn, may ultimately result in the selection BTZ-resistant clones. Although the exact basis of BTZ resistance is multifactorial and has not been fully elucidated, there are several mechanisms mediating this treatment-hindering phenomenon. BTZ is a proteasome inhibitor that selectively and reversibly blocks its chymotryptic site [30,37]. Therefore, upon exposure to BTZ, drug-induced environmental pressure causes MM cells to develop proteasome alterations that may impede the anti-MM activity of BTZ. Indeed, such a hypothesis was demonstrated to drive BTZ resistance in different models [38,39]. Another mechanism contributing to BTZ resistance is related to the bone marrow microenvironment and the interplay between proinflammatory macrophages and MM cells [40].

There is a shortage of studies investigating the role of epigenetic alterations in the pathophysiology of acquired BTZ resistance. Therefore, we previously investigated the molecular background of this phenomenon in various models. For instance, we demonstrated that changes in the methylome of neuroblastoma cells contribute to resistance to this compound. We performed repeatedly treated SH-SY5Y neuroblastoma cells with BTZ until resistance was acquired. Our results showed that BTZ induces methylation changes affecting the proliferative potential of neuroblastoma cells [22]. Using a similar methodology, we also investigated the mechanisms underlying BTZ resistance in MM. We established the BTZ-resistant U266 MM cell line by repeated coincubation with BTZ. Among others, we showed that SNORD-family genes were upregulated compared to control cells, suggesting the involvement of epigenetic mechanisms [23]. In another study, treatment of BTZ-resistant mantle cell lymphoma cells with BTZ and DNA methyltransferase inhibitor decitabine (DAC) resulted in the death of around 80% of cells. It should be noted that both BTZ and DAC monotherapy killed approximately 10–25% of BTZ-resistant mantle cell lymphoma cells [41]. Other studies have highlighted the role of long non-coding RNA in conferring BTZ resistance in MM [42,43].

Because SNORDs are involved in mediating epigenetic changes [44], we hypothesized that epigenetic alterations play a role in the development of BTZ resistance and, secondly, that epigenetic drugs such as 5-Aza-2′-deoxycytidine may potentially be beneficial in BTZ-resistant MM.

In the present study, based on our previous findings [22,23], we hypothesized that alterations in the methylation profile may mediate BTZ resistance and that epigenetic agents such as 5-Aza-2′-deoxycytidine may act synergistically with BTZ, similarly to VD and VK. We deepened our analysis and investigated the effect of a methylation inhibitor on the proliferative potential of MM cells and the development of BTZ resistance. In this paper, we demonstrated that alterations in the methylation profile are associated with BTZ resistance. First, our results revealed that the addition of methylation inhibitor 5-Aza-2′-deoxycytidine to BTZ-resistant MM cells led to a reduction in the proliferation of the BTZ-resistant phenotype, resulting in the restoration of sensitivity to BTZ. Moreover, a comparison of the global DNA methylation profile between the second and third treatments showed a continuous decrease in methylation. What should be emphasized is that no hypermethylation sites were found after the third treatment (BTZ_m i vs. BTZ_3). It can therefore be concluded that a decrease in methylation results in the restoration of chemosensitivity. Liu et al. demonstrated that BTZ induced global hypomethylation in acute myeloid leukemia cells both in vitro and in vivo [45]. Hence, it can be hypothesized that a good response to treatment is primarily associated with loss of methylation. Increased methylation may contribute to the development of a BTZ-resistant phenotype. The addition of a methylation inhibitor may act synergistically or, when added to resistant MM cells, restore sensitivity to BTZ. Heatmap analysis of selected genes after the second and third treatments revealed significant differences in methylation levels between BTZ_2 and BTZ_m_2 and between BTZ_3 (resistant cells) and BTZ_m_3. More precisely, the development of BTZ resistance is associated with an increase in methylation, while drug sensitivity is associated with hypomethylation. Similar phenomena have been observed by other researchers. For instance, in an in vitro study, Hu and colleagues demonstrated that CD9 downregulation by methylation decreased BTZ sensitivity in U266 MM cells. Moreover, they demonstrated that a methylation inhibitor, namely 5-Aza-2-deoxycytidine, upregulated CD9 and raised sensitivity to BTZ [46]. Due to the complex nature of interactions in the human body, the results obtained in in vitro studies provide only first insights and may not be entirely conclusive in terms of translation to clinical conditions. De Larrea and coworkers investigated the relationship between overall survival (OS), progression-free survival (PFS), and both global and gene-specific methylation profiles in MM patients treated with BTZ-based chemotherapy regimens. A proportion of 62% of the analyzed patients responded to treatment (complete remission, 6.7%; partial response, 44%; minimal response, 10.7%). Globally, they observed low methylation status across the entire cohort. Nevertheless, patients with more than 3.95% of total DNA methylated achieved better OS than patients with more unmethylated DNA (median of 30 versus 15 months; *p* = 0.004). On the other hand, they demonstrated that hypomethylation of the *NFKB1* and *CXCR4* genes was associated with a better response to treatment [47].

In addition to the trend that global DNA hypomethylation confers BTZ sensitivity, our heat maps we demonstrate the methylation status of selected genes, as well as various processes, according to GSEA analysis. We identified several genes that differed in methylation status between MM cells treated with BTZ alone and those incubated additionally with a methylation inhibitor. For instance, after second passage, we identified differences in methylation levels in the island area of the *ORAI3* gene, the overexpression of which has been linked to chemotherapy resistance in breast cancer [48]. Furthermore, the 5′UTR-opensea region of the *FBXW7* gene was markedly less methylated in BTZ-treated MM cells. This gene and, more specifically, its downregulation have also been shown to play a role in mediating drug resistance and chemotherapy response in cancers [49,50,51,52]. After the third passage, we once again identified hypomethylation of the *ORAI3* gene in MM cells incubated with BTZ and a methylation inhibitor (BTZ-sensitive cells) and hypermethylation in BTZ-resistant MM cells. Moreover, we identified differences in the methylation status of the *MIR21* gene, which is linked to chemotherapy resistance in various cancers, such as ovarian cancer [53] and renal carcinoma [54]. In addition, the *PRC1* gene was demonstrated to be associated with drug resistance and poor clinical outcomes in various malignancies [55,56,57].

GSEA analysis showed statistically significant changes in several key processes involved in the development of resistance. The most important seems to be RNA splicing. The influence of RNA splicing on the development of drug resistance has been demonstrated in many oncological diseases. In some cases of chronic myeloid leukemia (CML), expression of high levels of alternatively spliced BCR-ABL mRNA with a 35 bp insertion (35INS) between exons 8 and 9 of the ABL kinase domain has been observed. This insertion shifts the frame, leading to the addition of 10 residues and the truncation of 653 residues due to early termination. These changes provide resistance to imatinib, which depends on the level of expression [58]. In breast and ovarian cancer cells, a mutation was induced in exon 11 of BRCA1, expressing the BRCA1-Δ11q splice variant lacking most of exon 11. The introduction of the mutation resulted in a frameshift to exon 11. The nascent BRCA1-Δ11q protein was able to promote partial resistance to the PARP inhibitor (PARPi) and cisplatin compared to full-length BRCA1 both in vitro and in vivo [59].

We identified differences in the methylation status of various genes that have been demonstrated to play an important role in mediating chemotherapy resistance. However, it should be noted that the vast majority of them were hyper- or hypomethylated in regions other than the promoter. Therefore, one may presume that their methylation status does not necessarily accurately reflect expression. To draw more firm conclusions, expression should be assessed at the RNA and protein levels.

## 4. Materials and Methods

### 4.1. Cell Culture and Course of the Experiment

The U266 human multiple myeloma cell line (ATCC, Manassas, VA, USA) was used in this experiment. U266 cells were cultured using RPMI-1640 medium (ATCC, Manassas, VA, USA, cat no. 30-2001), which contained 2 mM L-glutamine, 10 mM HEPES, 1 mM sodium pyruvate, 4500 mg/L glucose, and 1500 mg/L sodium bicarbonate and was supplemented with 15% fetal bovine serum. The medium was changed every three days.

U266 cells were treated with BTZ at 2.75 nM (Cell Signaling Technology, Danvers, MA, USA) and methylation inhibitor 5-Aza-2′-deoxycytidine at 1 µM (Sigma Aldrich, St. Louis, MO, USA) three times for 24 h at 10-day intervals. The experiment was carried out in three technical repetitions. The dose of BTZ and a protocol for obtaining a resistant cell line were established in our previous article [23] and caused the death of over 50% of cells after the first treatment. The third treatment resulted in a fully BTZ-resistant cell phenotype. For this experiment, the concentration of 5-Aza-2′-deoxycytidine was determined by applying three experimental doses (10 nM, 100 nM, and 1000 nM) and performing a proliferation assay (results are presented in Section 2.1). DNA was isolated from the cells, and a proliferation assay was performed after each treatment, with some cells were left for further proliferation in medium without BTZ and 5-Aza-2′-deoxycytidine. DNA was also isolated from cells left for 10 days in a growth medium (free of BTZ and methylation inhibitor) after the third treatment to verify whether the changes in the methylation profile caused by 24 h incubation were permanent and transferred to the daughter cells.

### 4.2. DNA Extraction and Bisulfite Conversion

A PureLink Genomic DNA Mini Kit (Thermo Fisher, Waltham, MA, USA) was used for DNA isolation. Extraction was performed according to the manufacturer’s instructions. A Genomic DNA ScreenTape kit (Agilent Technologies, Santa Clara, CA, USA) was used to measure the concentration and quality of genetic material. The analysis was performed using TapeStation 4510 (Agilent Technologies, Santa Clara, CA, USA). For further procedures, samples showing DINs ≥ 9 were used, proving the good quality of the material. An EZ DNA Methylation-Gold Kit (Zymo Research, Irvine, CA, USA) was used for DNA conversion, with aims of deaminating unmethylated cytosines, resulting in uracil molecules in place of cytosines. Methylated cytosines do not react with sodium bisulfate and therefore remain unchanged after this reaction. All conversion procedures were carried out in accordance with the manufacturer’s recommendations. An amount of 500 ng of DNA from each sample was used for conversion.

### 4.3. Methylation Arrays

Changes in the DNA methylation profile were detected using a human Infinium Methylation EPIC BeadChip kit (Illumina, San Diego, CA, USA). Each technical replicate in the experiment was analyzed (*n* = 3). The procedure was performed strictly according to the array manufacturer’s instructions. A NextSeq550 instrument (Illumina, San Diego, CA, USA) was used to scan the arrays.

### 4.4. Dose Titration of 5-Aza-2′-Deoxycytidine

To determine the appropriate dose of methylation inhibitor, U266 cell proliferation analysis was performed at three doses of 5-Aza-2′-deoxycytidine (10 nM, 100 nM, and 1000 nM). Proliferation analysis was performed using an Alamar Blue mitochondrial dye conversion assay kit (Thermo Fisher, Waltham, MA, USA). The assay was performed in 96-well plates (1 × 10^4^ U266 cells/well). Each measurement was carried out in 8 wells. Proliferation-level analysis was performed after each of three 24 h treatments of cells with BTZ (control cell) (2.75 nM) or BTZ (2.75 nM) and methylation inhibitor (10 nM, 100 nM, and 1000 nM). After each treatment of U266 cells, proliferation analysis was performed 5 times (1, 3, 6, 8, and 10 days after administration).

### 4.5. Bioinformatics Analysis of Genome-Wide Methylation

Bioinformatics analyses were performed in the R programming environment using appropriate Bioconductor libraries. We screened for methylation changes using an Illumina Infinium Methylation EPIC Beadchip array. The array evaluated the methylation status of more than 850,000 CpG loci. Analyses were carried out in the R programming environment with the relevant Bioconductor libraries. The “ChAMP” pipeline was used to process the raw microarray data files (.idat) with the default processing options [60,61]. Probes meeting any of the following criteria were removed from the final dataset: detection *p*-value > 0.01, <3 beads in at least 5% of samples per probe, non-CpG probes, SNP-related probes, multihit probes, and probes located on chromosomes X and Y. Quality control and normalization of the obtained results were then performed. Batch effects and other unwanted variation were removed by the “sva” Bioconductor library [62]. A linear model from the “limma” package was employed to compute the adjusted p value and beta values (methylation scores for each CpG based on the fluorescent intensity, which varied between 0 (unmethylated) and 1 (completely methylated)) [63]. For each CpG probe, the delta beta value corresponding to differential methylation in compared groups was also calculated. A methylation difference with adjusted *p* < 0.05 and |delta beta| > 0.2 was considered statistically significant. The results were visualized using the following R libraries: “ggplot2”, “ggprism”, “ComplexHeatmap”, and “ggpubr” [62,63].

Significantly hypermethylated and hypomethylated probes associated with specific genes were analyzed separately for functional annotation and clustering using the DAVID bioinformatics tool [64,65]. The ENTREZIDs of the methylated genes were matched with relevant GO terms, and significantly enriched gene ontological (GO) terms were further selected from BP DIRECT’s GO database. Ontological groups with a corrected p value of less than 0.05 (after Benjamini–Hochberg correction) were visualized as bubble plots.

Gene set enrichment analysis (GSEA) was performed using the “clusterProfiler” library [27] to determine the level of depletion or enrichment in GO terms by calculating a normalized enrichment score (NES) with a corresponding p value. Delta beta values were sorted and used as an argument for the “gseGO” function. Enrichment of gene sets was performed for the GO category of “biological process,” assuming that the minimum size of each gene set for analysis was 50, with a p-value cutoff of 0.05. The ten ontology groups with the highest enrichment scores (highest NES values) and those with the most depleted enrichment scores (lowest NES values) were visualized as a bar chart. Enrichment charts for the five most enriched and depleted GO terms were also presented.

## 5. Conclusions

Changes in DNA methylation influence the development of the BTZ resistance phenotype, and an attempt to limit them by using a methylation inhibitor resulted in a significant reduction in the development of this phenotype. The conducted study may indicate the direction of further clinical trials aimed at modifying existing treatment regimens or developing new ones by including a methylation inhibitor, which can significantly reduce/eliminate the common problem of developing resistance to chemotherapy in patients with MM.

## 6. Study Limitations

Our study revealed interesting results that may provide a molecular basis for future clinical trials and contribute to improving the clinical outcomes of MM patients. Nevertheless, it has some drawbacks. First, this was an in vitro study. Laboratory conditions do not always accurately reflect the complexity of interactions occurring in the human organism. Therefore, a similar experiment should be performed in an animal model and, ultimately, in a clinical trial. The second disadvantage is that this study was conducted on a single cell line. Repeating the experiment in different MM cell lines and obtaining similar results would strengthen our conclusions and provide stronger evidence to support our hypothesis.

## Data Availability

Data are available from the corresponding author upon reasonable request.

## References

[1] Huang J., Chan S.C., Lok V., Zhang L., Lucero-Prisno D.E., Xu W., Zheng Z.J., Elcarte E., Withers M., Wong M.C.S. (2022). The epidemiological landscape of multiple myeloma: A global cancer registry estimate of disease burden, risk factors, and temporal trends. Lancet Haematol..

[2] Diamond E., Lahoud O.B., Landau H. (2018). Managing multiple myeloma in elderly patients. Leuk. Lymphoma.

[3] Jurczyszyn A., Suska A. (2019). Multiple Myeloma. Reference Module in Biomedical Sciences.

[4] Rajkumar S.V. (2020). Multiple myeloma: 2020 update on diagnosis, risk-stratification and management. Am. J. Hematol..

[5] Firth J., Medical Masterclass Contributors (2019). Haematology: Multiple myeloma. Clin. Med..

[6] Dhodapkar M.V. (2016). MGUS to myeloma: A mysterious gammopathy of underexplored significance. Blood.

[7] Kyle R.A., Therneau T.M., Rajkumar S.V., Offord J.R., Larson D.R., Plevak M.F., Melton 3rd L.J. (2002). A Long-Term Study of Prognosis in Monoclonal Gammopathy of Undetermined Significance. N. Engl. J. Med..

[8] Kyle R.A., Remstein E.D., Therneau T.M., Dispenzieri A., Kurtin P.J., Hodnefield J.M., Larson D.R., Plevak M.F., Jelinek D.F., Fonseca R. (2007). Clinical Course and Prognosis of Smoldering (Asymptomatic) Multiple Myeloma. N. Engl. J. Med..

[9] Rajkumar S.V., Kumar S. (2016). Multiple Myeloma: Diagnosis and Treatment. Mayo Clin. Proc..

[10] Rajkumar S.V., Kumar S. (2020). Multiple myeloma current treatment algorithms. Blood Cancer J..

[11] Koeppen S. (2014). Treatment of Multiple Myeloma: Thalidomide-, Bortezomib-, and Lenalidomide-Induced Peripheral Neuropathy. Oncol. Res. Treat..

[12] Richardson P.G., Sonneveld P., Schuster M., Irwin D., Stadtmauer E., Facon T., Harousseau J.-L., Ben-Yehuda D., Lonial S., Goldschmidt H. (2007). Extended follow-up of a phase 3 trial in relapsed multiple myeloma: Final time-to-event results of the APEX trial. Blood.

[13] Soave C.L., Guerin T., Liu J., Dou Q.P. (2017). Targeting the ubiquitin-proteasome system for cancer treatment: Discovering novel inhibitors from nature and drug repurposing. Cancer Metastasis Rev..

[14] Haeri M., Knox B.E. (2012). Endoplasmic Reticulum Stress and Unfolded Protein Response Pathways: Potential for Treating Age-related Retinal Degeneration. J. Ophthalmic Vis. Res..

[15] Adams J., Palombella V.J., Sausville E.A., Johnson J., Destree A., Lazarus D.D., Maas J., Pien C.S., Prakash S., Elliott P.J. (1999). Proteasome inhibitors: A novel class of potent and effective antitumor agents. Cancer Res..

[16] Handy D.E., Castro R., Loscalzo J. (2011). Epigenetic Modifications: Basic Mechanisms and Role in Cardiovascular Disease. Circulation.

[17] Holoch D., Moazed D. (2015). RNA-mediated epigenetic regulation of gene expression. Nat. Rev. Genet..

[18] Zhang P., Torres K., Liu X., Liu C., Pollock R.E. (2016). An Overview of Chromatin-Regulating Proteins in Cells. Curr. Protein Pept. Sci..

[19] Palomeras S., Diaz-Lagares Á., Viñas G., Setien F., Ferreira H.J., Oliveras G., Crujeiras A.B., Hernández A., Lum D.H., Welm A.L. (2019). Epigenetic silencing of TGFBI confers resistance to trastuzumab in human breast cancer. Breast Cancer Res..

[20] Haertle L., Barrio S., Munawar U., Han S., Zhou X., Vogt C., Fernández R.A., Bittrich M., Ruiz-Heredia Y., Da Viá M. (2021). Cereblon enhancer methylation and IMiD resistance in multiple myeloma. Blood.

[21] Che F., Chen J., Dai J., Liu X. (2020). Inhibition of Multiple Myeloma Using 5-Aza-2ʹ-Deoxycytidine and Bortezomib-Loaded Self-Assembling Nanoparticles. Cancer Manag. Res..

[22] Łuczkowska K., Sokolowska K.E., Taryma-Lesniak O., Pastuszak K., Supernat A., Bybjerg-Grauholm J., Hansen L.L., Paczkowska E., Wojdacz T.K., Machaliński B. (2021). Bortezomib induces methylation changes in neuroblastoma cells that appear to play a significant role in resistance development to this compound. Sci. Rep..

[23] Łuczkowska K., Kulig P., Baumert B., Machaliński B. (2022). The Evidence That 25(OH)D3 and VK2 MK-7 Vitamins Influence the Proliferative Potential and Gene Expression Profiles of Multiple Myeloma Cells and the Development of Resistance to Bortezomib. Nutrients.

[24] Moore L.D., Le T., Fan G. (2013). DNA Methylation and Its Basic Function. Neuropsychopharmacology.

[25] Romero-Garcia S., Prado-Garcia H., Carlos-Reyes A. (2020). Role of DNA Methylation in the Resistance to Therapy in Solid Tumors. Front. Oncol..

[26] Cao Y., Qiu G.-Q., Wu H.-Q., Wang Z.-L., Lin Y., Wu W., Xie X.-B., Gu W.-Y. (2016). Decitabine enhances bortezomib treatment in RPMI 8226 multiple myeloma cells. Mol. Med. Rep..

[27] Wu T., Hu E., Xu S., Chen M., Guo P., Dai Z., Feng T., Zhou L., Tang W., Zhan L. (2021). clusterProfiler 4.0: A universal enrichment tool for interpreting omics data. Innovation.

[28] Wang B.-D., Lee N.H. (2018). Aberrant RNA Splicing in Cancer and Drug Resistance. Cancers.

[29] Salehan M.R., Morse H.R. (2013). DNA damage repair and tolerance: A role in chemotherapeutic drug resistance. Br. J. Biomed. Sci..

[30] Richardson P.G., Barlogie B., Berenson J., Singhal S., Jagannath S., Irwin D., Rajkumar S.V., Srkalovic G., Alsina M., Alexanian R. (2003). A Phase 2 Study of Bortezomib in Relapsed, Refractory Myeloma. N. Engl. J. Med..

[31] Palumbo A., Chanan-Khan A., Weisel K., Nooka A.K., Masszi T., Beksac M., Spicka I., Hungria V., Munder M., Mateos M.V. (2016). Daratumumab, Bortezomib, and Dexamethasone for Multiple Myeloma. N. Engl. J. Med..

[32] Mateos M.-V., Sonneveld P., Hungria V., Nooka A.K., Estell J.A., Barreto W., Corradini P., Min C.K., Medvedova E., Weisel K. (2020). Daratumumab, Bortezomib, and Dexamethasone Versus Bortezomib and Dexamethasone in Patients With Previously Treated Multiple Myeloma: Three-year Follow-up of CASTOR. Clin. Lymphoma Myeloma Leuk..

[33] Rosiñol L., Oriol A., Teruel A.I., Hernández D., López-Jiménez J., De La Rubia J., Granell M., Besalduch J., Palomera L., González Y. (2012). Superiority of bortezomib, thalidomide, and dexamethasone (VTD) as induction pretransplantation therapy in multiple myeloma: A randomized phase 3 PETHEMA/GEM study. Blood.

[34] Rosiñol L., Oriol A., Rios R., Sureda A., Blanchard M.J., Hernández M.T., Martínez-Martínez R., Moraleda J.M., Jarque I., Bargay J. (2019). Bortezomib, lenalidomide, and dexamethasone as induction therapy prior to autologous transplant in multiple myeloma. Blood.

[35] Richardson P.G., Oriol A., Beksac M., Liberati A.M., Galli M., Schjesvold F., Lindsay J., Weisel K., White D., Facon T. (2019). Pomalidomide, bortezomib, and dexamethasone for patients with relapsed or refractory multiple myeloma previously treated with lenalidomide (OPTIMISMM): A randomised, open-label, phase 3 trial. Lancet Oncol..

[36] Moreau P., Attal M., Hulin C., Arnulf B., Belhadj K., Benboubker L., Béné M.C., Broijl A., Caillon H., Caillot D. (2019). Bortezomib, thalidomide, and dexamethasone with or without daratumumab before and after autologous stem-cell transplantation for newly diagnosed multiple myeloma (CASSIOPEIA): A randomised, open-label, phase 3 study. Lancet.

[37] Richardson P.G., Mitsiades C., Hideshima T., Anderson K.C. (2006). Bortezomib: Proteasome Inhibition as an Effective Anticancer Therapy. Annu. Rev. Med..

[38] Oerlemans R., Franke N.E., Assaraf Y.G., Cloos J., Van Zantwijk I., Berkers C.R., Scheffer G.L., Debipersad K., Vojtekova K., Lemos C. (2008). Molecular basis of bortezomib resistance: Proteasome subunit β5 (PSMB5) gene mutation and overexpression of PSMB5 protein. Blood.

[39] Wu Y.-X., Yang J.-H., Saitsu H. (2016). Bortezomib-resistance is associated with increased levels of proteasome subunits and apoptosis-avoidance. Oncotarget.

[40] Beyar-Katz O., Magidey K., Reiner-Benaim A., Barak N., Avivi I., Cohen Y., Timaner M., Avraham S., Hayun M., Lavi N. (2019). Proinflammatory Macrophages Promote Multiple Myeloma Resistance to Bortezomib Therapy. Mol. Cancer Res..

[41] Leshchenko V.V., Kuo P.-Y., Jiang Z., Weniger M.A., Overbey J., Dunleavy K., Wilson W.H., Wiestner A., Parekh S. (2015). Harnessing Noxa demethylation to overcome Bortezomib resistance in mantle cell lymphoma. Oncotarget.

[42] Yang L.-H., Du P., Liu W., An L.-K., Li J., Zhu W.-Y., Yuan S., Wang L., Zang L. (2021). LncRNA ANRIL promotes multiple myeloma progression and bortezomib resistance by EZH2-mediated epigenetically silencing of PTEN. Neoplasma.

[43] Tang J., Chen Q., Li Q., He Y., Xiao D. (2021). Exosomal mRNAs and lncRNAs involved in multiple myeloma resistance to bortezomib. Cell Biol. Int..

[44] Watkins N.J., Bohnsack M.T. (2012). The box C/D and H/ACA snoRNPs: Key players in the modification, processing and the dynamic folding of ribosomal RNA. WIREs RNA.

[45] Liu S., Liu Z., Xie Z., Pang J., Yu J., Lehmann E., Huynh L., Vukosavljevic T., Takeki M., Klisovic R.B. (2008). Bortezomib induces DNA hypomethylation and silenced gene transcription by interfering with Sp1/NF-κB–dependent DNA methyltransferase activity in acute myeloid leukemia. Blood.

[46] Hu X., Xuan H., Du H., Jiang H., Huang J. (2014). Down-Regulation of CD9 by Methylation Decreased Bortezomib Sensitivity in Multiple Myeloma. Agoulnik IU, editor. PLoS ONE.

[47] Fernández De Larrea C., Martín-Antonio B., Cibeira M.T., Navarro A., Tovar N., Díaz T., Rosiñol L., Monzó M., Urbano-Ispizua A., Bladé J. (2013). Impact of global and gene-specific DNA methylation pattern in relapsed multiple myeloma patients treated with bortezomib. Leuk. Res..

[48] Hasna J., Hague F., Rodat-Despoix L., Geerts D., Leroy C., Tulasne D., Ouadid-Ahidouch H., Kischel P. (2018). Orai3 calcium channel and resistance to chemotherapy in breast cancer cells: The p53 connection. Cell Death Differ..

[49] Fan J., Bellon M., Ju M., Zhao L., Wei M., Fu L., Nicot C. (2022). Clinical significance of FBXW7 loss of function in human cancers. Mol. Cancer.

[50] Chen S., Lin J., Zhao J., Lin Q., Liu J., Wang Q., Mui R., Ma L. (2023). FBXW7 attenuates tumor drug resistance and enhances the efficacy of immunotherapy. Front. Oncol..

[51] Mun G.-I., Choi E., Lee Y., Lee Y.-S. (2020). Decreased expression of FBXW7 by ERK1/2 activation in drug-resistant cancer cells confers transcriptional activation of MDR1 by suppression of ubiquitin degradation of HSF1. Cell Death Dis..

[52] Wang Z., Fukushima H., Gao D., Inuzuka H., Wan L., Lau A.W., Liu P., Wei W. (2011). The two faces of FBW7 in cancer drug resistance. BioEssays.

[53] Sheng S., Su W., Mao D., Li C., Hu X., Deng W., Yao Y., Ji Y. (2022). MicroRNA-21 induces cisplatin resistance in head and neck squamous cell carcinoma. PLoS ONE.

[54] Gaudelot K., Gibier J.-B., Pottier N., Hémon B., Van Seuningen I., Glowacki F., Leroy X., Cauffiez C., Gnemmi V., Aubert S. (2017). Targeting miR-21 decreases expression of multi-drug resistant genes and promotes chemosensitivity of renal carcinoma. Tumor Biol..

[55] Bu H., Li Y., Jin C., Yu H., Wang X., Chen J., Wang Y., Ma Y., Zhang Y., Kong B. (2020). Overexpression of PRC1 indicates a poor prognosis in ovarian cancer. Int. J. Oncol..

[56] Parreno V., Martinez A.-M., Cavalli G. (2022). Mechanisms of Polycomb group protein function in cancer. Cell Res..

[57] Liang Z., Li X., Chen J., Cai H., Zhang L., Li C., Tong J., Hu W. (2019). PRC1 promotes cell proliferation and cell cycle progression by regulating p21/p27-pRB family molecules and FAK-paxillin pathway in non-small cell lung cancer. Transl. Cancer Res..

[58] Lee T.-S., Ma W., Zhang X., Giles F., Cortes J., Kantarjian H., Albitar M. (2008). BCR-ABL alternative splicing as a common mechanism for imatinib resistance: Evidence from molecular dynamics simulations. Mol. Cancer Ther..

[59] Wang Y., Bernhardy A.J., Cruz C., Krais J.J., Nacson J., Nicolas E., Peri S., van der Gulden H., van der Heijden I., O’Brien S.W. (2016). The BRCA1-Δ11q Alternative Splice Isoform Bypasses Germline Mutations and Promotes Therapeutic Resistance to PARP Inhibition and Cisplatin. Cancer Res..

[60] Tian Y., Morris T.J., Webster A.P., Yang Z., Beck S., Feber A., Teschendorff A.E. (2017). ChAMP: Updated methylation analysis pipeline for Illumina BeadChips. Bioinformatics.

[61] Morris T.J., Butcher L.M., Feber A., Teschendorff A.E., Chakravarthy A.R., Wojdacz T.K., Beck S. (2014). ChAMP: 450k Chip Analysis Methylation Pipeline. Bioinformatics.

[62] Leek J.T., Evan Johnson W., Parker H.S., Fertig E.J., Jaffe A.E., Zhang Y., Storey J.D., Collado Torres L. (2021). Sva: Surrogate Variable Analysis.

[63] Ritchie M.E., Phipson B., Wu D., Hu Y., Law C.W., Shi W., Smyth G.K. (2015). limma powers differential expression analyses for RNA-sequencing and microarray studies. Nucleic Acids Res..

[64] Dennis G., Sherman B.T., Hosack D.A., Yang J., Gao W., Lane H.C., Lempicki R.A. (2003). DAVID: Database for Annotation, Visualization, and Integrated Discovery. Genome Biol..

[65] Fresno C., Fernández E.A. (2013). RDAVIDWebService: A versatile R interface to DAVID. Bioinformatics.

